# Aging of the Hair Follicle Pigmentation System

**DOI:** 10.4103/0974-7753.58550

**Published:** 2009

**Authors:** Desmond J Tobin

**Affiliations:** Centre for Skin Sciences, School of Life Sciences, University of Bradford, Bradford, West Yorkshire, Great Britain

**Keywords:** Hair follicle, melanocyte, reactive oxygen species

## Abstract

Skin and hair phenotypes are powerful cues in human communication. They impart much information, not least about our racial, ethnic, health, gender and age status. In the case of the latter parameter, we experience significant change in pigmentation in our journey from birth to puberty and through to young adulthood, middle age and beyond. The hair follicle pigmentary unit is perhaps one of our most visible, accessible and potent aging sensors, with marked dilution of pigment intensity occurring long before even subtle changes are seen in the epidermis. This dichotomy is of interest as both skin compartments contain melanocyte subpopulations of similar embryologic (i.e., neural crest) origin. Research groups are actively pursuing the study of the differential aging of melanocytes in the hair bulb versus the epidermis and in particular are examining whether this is in part linked to the stringent coupling of follicular melanocytes to the hair growth cycle. Whether some follicular melanocyte subpopulations are affected, like epidermal melanocytes, by UV irradiation is not yet clear. A particular target of research into hair graying or canities is the nature of the melanocyte stem compartment and whether this is depleted due to reactive oxygen species-associated damage, coupled with an impaired antioxidant status, and a failure of melanocyte stem cell renewal. Over the last few years, we and others have developed advanced *in vitro* models and assay systems for isolated hair follicle melanocytes and for intact anagen hair follicle organ culture which may provide research tools to elucidate the regulatory mechanisms of hair follicle pigmentation. Long term, it may be feasible to develop strategies to modulate some of these aging-associated changes in the hair follicle that impinge particularly on the melanocyte populations.

## INTRODUCTION

Skin contains at least 3 pigment classes, including hemoglobin, carotenoids and melanin pigments.[1] However, only the melanins make a significant contribution to our overall skin and hair color. These represent a class of mixed indole-rich compounds and are produced in a phylogenetically ancient and complex biochemical pathway termed *melanogenesis*. This process occurs in unique lysosome-related organelles called *melanosomes*, whose biogenesis, maturation and trafficking occurs within the cytoplasm of cells called *melanocytes*. Skin pigmentation and hair growth/pigmentation have clearly facilitated evolutionary success in other mammals via thermoregulation, camouflage and trauma protection, for example. However, it has been much more difficult to determine how hair growth and pigmentation played a fundamental role in human survival over time. Still, as much of the human species' success is due to its powerful social strategies, it is reasonable to assume that skin and hair color would contribute significantly in human social communications as these traits provide much information regarding an individual's race, ethnicity, gender, age and health status, as well as physical and sexual attractiveness. Just why human scalp hair, at least in youth, is so luxuriant and pigmented is unclear but may have had an important evolutionary selective pressure,[2‐5] but one specific to humans as this trait is unique among the primates.

Scalp hair follicles contain some of the most highly proliferative tissues in the human body, with a cell division rate second only to the bone marrow and gut epithelium.[6] Indeed, almost 100% of keratinocytes in the growing (anagen) hair bulb are cycling at any one time and can continue to do so for an extraordinarily long period of time. In some cases, there may be a block in anagen whereby scalp hair grows to lengths in excess of 4 meters of fully pigmented fiber. To generate a hair fiber of such a length, the hair follicle would have to be in a continuous anagen for 25 or more years. One particularly famous example is that of a 42-year-old Chinese woman.[7] More usually, anagen persists for 3 to 5 years, and these follicles extrude the hair fiber at a rate of approximately 1 cm per month.[6] The fact that these extra-long fibers can be fully pigmented attests to the enormous potential for melanogenic activity invested in growing pigmented scalp hair. Moreover, the selective and avid binding of toxins and metals to melanin pigment[3,4,8]supports the view that melanin could act as an important sink for sequestering potentially noxious materials, thereby limiting their access (and so potential damage) to the living tissue of the highly vascularized scalp.

The importance of eumelanin (brown/black) cannot be understated, given its predominance in global skin and hair pigmentation distribution. The vast majority of the global human population has dark brown/black scalp hair during their early/mid lives, reflecting our out-of-Africa origins and the climatic imperatives associated with this.[9] However, what about the remaining ~5% of humans concentrated today in northern Europe who have emerged with a remarkably diverse palette of colors, e.g., white blonde, yellow blonde, red, auburn and all shades in between? The discovery that a limited range of mutations in the melanocortin-1 receptor (MC1-R) gene can underlie this pheomelanic variety has been a significant achievement.[10,11] Indeed, this G protein-coupled receptor has contributed hugely to our understanding of mammalian pigmentation,[10,11] as the MC1-R is activated, in the main, by pro-melanogenic peptides ligands derived from pro-opiomelanocortin, including α-melanocyte-stimulating hormone (α-MSH) and adrenocorticotropin hormone (ACTH).[9,10] Some subpopulations of northwestern Europeans, especially those with red hair, are homozygotes or compound heterozygotes for a limited number of MC1-R mutations.[11]

Here I will provide a brief review of the regulation of hair follicle pigmentation before focusing instead on our current knowledge of the aging pigmentary unit and canities (or hair graying). Interested readers are directed to recent more comprehensive reviews on the topic.[5,12‐14]

## DEVELOPMENT OF THE HAIR FOLLICLE PIGMENTATION UNITS

The life history of a cutaneous melanocyte, after its lineage commitment in the neural crest during embryogenesis, is an eventful and protracted one and involves relatively quiescent stem cells, proliferating, differentiating, senescencing stages followed by cell death. Melanocytes of both the epidermal and follicular melanin units are derived from immature 'melanoblasts' that migrate from the neural crest into the skin during embryogenesis. Just how some neural crest cells are committed to the melanocyte lineage remains a subject of intense interest for research,[15,16]and so far it appears that microphthalmia-associated transcription factor (MITF), SRY-related HMG-BOX10 (SOX10), Paired Box 3 (Pax 3), KIT, fibroblast growth factor-2, and endothelin-3 are all involved to variable extents.[16] Once committed, differentiation of the early melanoblasts appears to require endothelin-3 for their onward journey to the epidermis,[17] so that by 7 weeks of human gestation we can see initiation of melanin production (melanogenesis) even before these cells engage with the now developing hair follicle.[18] Recent research suggests that immature melanocytes even in adult mammalian skin may retain some plasticity regarding their potential differentiation trajectories.[16]

As the hair follicle develops the progeny of melanoblasts/melanocytes which proliferate in the epidermis (often called 'transit or transient-amplifying' melanocytes), leave that compartment and move into the developing hair follicle. There, melanocytes may become/remain dopa-oxidase-positive cells (i.e., express active tyrosinase) or remain dopa-oxidase-negative cells (i.e., either fail to express tyrosinase or express an inactive tyrosinase) depending on the intra-follicular compartment in which they reside.[19,20] Such compartmentalization of follicular melanocyte subpopulations during skin development is likely to be very important for considerations of melanocyte 'renewal' during the hair growth cycle, the role of the stem melanocyte reservoir, and during age-related pigmentation changes and age-related depletion of functioning melanocytes during graying.

## DIVERSITY OF MELANOCYTE SUBPOPULATIONS IN SKIN AND HAIR FOLLICLE: IMPLICATIONS FOR HAIR AGING

While observing each other, we can soon appreciate the apparent independence of the epidermal and follicular melanin units in those with both white hair and black skin, for example in aging people of African descent. Conversely, in Irish or Scottish populations, it is not uncommon to see blue-eyed individuals with raven black hair but very pale and freckle-free skin. Ultrastructural examination of melanocytes in both the epidermis and hair bulb compartments reveals some striking differences between these two closely related melanocyte populations. Differentiated hair bulb melanocytes tend to be larger, more dendritic, have more extensive Golgi and rough endoplasmic reticulum and produce larger melanosomes than their epidermal cousins.[15,21] Moreover, while melanin granules are almost completely degraded in the differentiating layers of the epidermis, melanin granules transferred into pre-cortical keratinocytes in the hair follicle remain minimally digested (though red/yellow pheomelanin appears to be less resistant to degradation than black/ brown eumelanin) as they become locked into the hair fiber from proximal to distal tip.

Follicular melanocytes distributed superficially in the hair follicle at the level of the infundibulum are melanogenically active (i.e., with 3, 4-dihydroxy phenylalanine dopa-oxidase activity of tyrosinase) as are melanocytes commonly found located between sebocytes in the basal layer of the sebaceous gland. It is not yet clear what role the latter melanocyte subpopulation has, though this is likely to be at least in part antimicrobial in this hormone-sensitive holocrine gland.[22] Proximal to these melanocyte subpopulations are scattered dopa oxidase-negative and so amelanotic melanocytes of the mid-to-lower outer root sheath. These follicular pigment cells may represent a pool of "transient" melanocytes that migrate from precursor melanocyte stores in the hair follicle bulge to other areas of the outer root sheath.[23]

The hair bulb matrix is the principal site for the fully differentiated follicular melanocyte subpopulation; these melanocytes are distributed, in particular, within the matrix above and around the upper dermal papilla. These differentiated cells (perhaps including even some terminally differentiated) transfer their melanin granules to keratinocytes of the hair cortex and less so to the medulla and very rarely to the hair cuticle. However, we have shown that the transient lower hair follicle and bulb also contain an enigmatic additional but minor subpopulation of poorly differentiated melanocytes[24] distributed to the most proximal and peripheral regions of the growing hair bulb. The true function of this minor population is unclear at this stage, but it may represent a migratory population that maintains bulbar complement of functioning melanocytes or it may indeed have a nonpigmentary role.[25] We and others have attempted to characterize these multiple subpopulations of melanocytes using *in vitro* strategies. While immature follicular melanocytes can be detected in primary cultures of follicular melanocytes, it is not yet clear whether some of these cells may indeed have *bona fide* melanoblast or stem cell potential.[26]

## DOES CONTINUAL HAIR FOLLICLE CYCLING HAVE IMPLICATIONS FOR AGING OF THE HAIR PIGMENTARY UNIT?

While epidermal and follicular melanocytes may share a common origin and these compartments, while distinct, may remain open under certain circumstances (e.g., repigmentation of epidermis from upper hair follicle in vitiligo),[27] their biology is regulated very differently.[28‐30] The follicular melanocyte engages in episodic activity driven by hair cycle changes, while epidermal melanocyte activity remains broadly continuous. The constitutive activity of the latter can be stimulated further in a facultative manner, for example by UV irradiation. Active hair follicle pigmentation occurs only during active hair growth (i.e., anagen), and one would imagine that the duration of this anagen phase could have direct implications for follicular melanocyte homeostasis. Thus, while some scalp hair follicles bulbar melanocytes will be engaged in melanogenesis for up to 10 years, active melanogenesis of less than 1 month is likely in eyebrow hair follicles. However, so-called blocked-in-anagen cases (see above) force us to conclude that there may be enormous melanocyte capacity in the hair follicle pigmentary unit during a single (albeit 26 years long) anagen phase.[7] This view is based, however, on the unproven supposition that there is little or no replacement of bulbar melanocytes during a single anagen phase. Our recent identification of a second, immature melanocyte subpopulation in the proximal bulb leaves open the possibility for intra-anagen turnover of terminally differentiated bulbar melanocytes.

No melanin pigment is actively produced (tyrosinase and tyrosinase-related protein 1 mRNA and protein are also undetectable) during telogen, the relative-resting stage of the hair cycle. This runt stage of the hair cycle consists of less that 30% of the growing hair follicles in terms of tissue mass but still contains all cell precursors needed to reconstitute a fully pigmented growing anagen hair follicle. Whatever factor or factors that trigger the resting telogen hair follicle into early anagen may also be responsible for the reactivation of the follicular pigmentary unit. Some of these early melanocytes/melanoblasts express (at least in mouse hair follicles) dopachrome tautomerase, and a subpopulation of these express the melanocyte-survival marker KIT.[31] When the new anagen is being established in earnest, the differentiating melanocytes then begin to express tyrosinase mRNA and subsequently tyrosinase protein but not yet in its inactive form, along with tyrosinase receptor protein (TRP1). Melanocytes that will remain undifferentiated by residing in the upper outer root sheath (site of the presumptive germ cell reservoir) will remain TRP1 negative, while the subpopulation of melanocytes destined for the hair new bulb (TRP1/DCT/KIT-positive) will enter a phase of proliferation[17] and differentiation.

As with any complex tissue, a critical determinant of melanocyte behavior is the nature of its local microenvironment. This likely explains the striking observation that only follicular melanocytes located close to the anagen follicular papilla are in a permissive location for melanogenesis. This does not appear to involve a generic or shared mesenchymal signal *per se*, as melanocytes in the outer root sheath closely associated with mesenchyme, i.e., the dermal or connective tissue sheath, remain for the most part amelanotic.[19,20] Thus, it is likely that the follicular papilla cells exhibit a specific regulatory biochemical milieu that favors induction melanogenesis, perhaps via the production of high amounts of l-tyrosine from l-phenylalanine.[32,33] Moreover, a particular redox environment appears to exist in the follicular papilla to support melanogenesis; perhaps to be expected given that reactive oxygen species (ROS) are themselves produced as a function of melanogenesis.[32] This latter point has implications for canities, as oxidative stress may play an important role in hair graying.[4,21,33]

The transition between full anagen and the start of catagen-associated regression of the hair follicle during the adult hair cycle is a subject of intense research. It is during this phase of the cycle that we see such different biology for follicular melanocyte subpopulations and melanocytes of the epidermal melanin unit. For example, the activity and expression levels of tyrosinase decrease rapidly to become undetectable/very low in late catagen.[28,29] This physiologic decrease in follicular melanogenesis during catagen may reflect a general withdrawal of growth- and differentiation-supporting morphogens and mitogens from the entire transient hair follicle or the more specific exhaustion of an active signaling system that stimulates melanogenesis. Alternatively (or additionally) this change could involve the production of inhibitors of melanocyte activity. A clue for at least a partial involvement of the latter is that changes in the melanocyte predate final cessation of keratinocyte proliferation, with early melanocyte effects, including retraction of cell dendrites.[34] In this context, one may view this melanocyte deletion event as a brief 'canities-like' process of unknown function at the end of each hair cycle. This limited amount of keratinocyte proliferation continues for a short while causing the proximal section of hair shaft to remain unpigmented. Equally curious is the production of pigment at the end of anagen that is not incorporated into the hair shaft but is instead distributed to the follicular papilla, epithelial strand or connective tissue sheath of these regressing catagen hair follicles. Similar pigment incontinence is also seen during canities, again highlighting some similarities between hair bulb melanocyte fate during catagen and canities (see below).

A further change in the redox status of the regressing hair follicle is associated with a marked reduction in DCT activity during catagen, and this tautomerase is thought to improve melanocyte survival to oxidative stress.[35] Moreover, pterin (e.g., 6BH 4 ) synthesis and PAH activities are at their lowest during catagen, which along with rising levels of thioredoxin reductase provide for a reducing environment that is not conducive to melanogenesis. This reduces the supply of L-tyrosine to the hair follicle pigmentary unit, further generating conditions unfavorable for active melanogenesis.[32]

Formal evidence of the disappearance of mature hair bulb melanocytes during catagen has recently been forthcoming.[34,36] Before this, the prevailing view was that the hair bulb melanocyte system was a self-perpetuating system, whereby melanocytes involved in the pigmentation of one hair generation remain involved in the pigmentation of the next generation and so in the resisted catagen-driven apoptosis in the hair bulb.[37] Our current view is, by contrast, that melanocyte replacement must occur from the melanocyte reservoir in the upper hair follicle[34] and that this parallels the loss of at least a proportion of the mostly highly melanotic (and possibly terminally differentiated) hair bulb melanocytes by apoptosis.[36] It is possible, however, that some melanogenically active melanocytes derive from a subpopulation of catagen-surviving melanocytes,[37] though these may themselves be lost via apoptosis in the next hair cycle changeover. This view of melanocyte hair cycle-associated dynamics necessitates the existence of a flexible hair follicle melanocyte "stem" reservoir in the permanent part of the cycling hair follicle.[23]

## AGING OF MELANOCYTES OF THE FOLLICLE PIGMENTARY UNIT

A single hair follicle can produce a range of different hair follicle types during its life history — from morphogenesis in the womb to our last living moments in old age. A fine (un)pigmented lanugo hair appears during fetal life, thereafter changing to a shorter unpigmented vellus hair during childhood. This is followed by an increase in size to less fine pigmented intermediate hair in the prepubescent child. At puberty these same follicles begin to produce longer thicker and most pigmented terminal hair shafts characteristic of the adult. Perhaps ironically, miniaturization of hair fibers (e.g., in androgenetic alopecia) may represent a partial reversal of this sequence later in life.[38]

The hair follicle pigmentary unit also is highly susceptible to age-related change, and this is especially visible in those of European ancestry. Broadly speaking hair color is at its lightest in early childhood and becomes progressively darker even before puberty and darkening further during adolescence and young adulthood[2,39] until the onset of hair graying or canities. Hair color darkening with age is largely due to the influence of hormones, including the sex steroid androgens and estrogens. We and others have also shown that melanocytes are exquisitely sensitive to (neuro)endocrine factors.[24] Regulation of hair color exhibits significant variations in different body regions, which may reflect significant local variations in hormonal stimulation of hair follicles — these too can become more visibly expressed at certain ages. A striking example of this effect is the phenomenon of hair heterochromia (i.e., more than one dominant shade of hair color across body sites), which also becomes more apparent with age. This phenomenon is most strikingly apparent for scalp and beard (e.g., brown scalp and red beard) but may also affect the scalp alone.[40]

Thirty years of age is a useful time point to operationally assess the effects of chronologic skin aging in humans- perhaps reflective of the period beyond which change is very unlikely to exert significant evolutionary (i.e., reproductive age) effect. Based on this consideration alone, it is difficult to predict any functional advantage for gray or white hair, beyond some social and communication signaling value.

The stable (i.e., constitutively active) epidermal melanin unit succumbs to a 10% to 20% reduction in pigment-producing epidermal melanocytes (whether in sun-exposed or unexposed skin) for every decade after 30 years of age.[41,42] Given the extremely low turnover of melanocytes in the epidermis (even after UV exposure), this would appear to indicate that epidermal melanocytes are relatively long-living cells. Contributing to this is the relatively high expression of Bcl2 in epidermal melanocytes, which may enable these cells to survive exogenous stressors, including UVR-induced reactive oxygen species (ROS), and the endogenous oxidative stress generated during melanogenesis itself. How melanocytes age and whether they age differently from other cell types is of interest. Most of the available data on this topic derives from *in vitro* studies, where a form of enforced proliferative state and replicative senescence is induced. Thus, we need to be cautious in our extrapolation of these data to melanocytes *in situ*.

### Molecular biology of melanocyte aging

The study of melanocyte aging[43,44] has been further stimulated by the significant recent interest in melanocyte stem cells — particularly how these cells may play a role in both hair graying and melanoma.[21,45] Melanocytes are lost with age not only from the skin (epidermis and hair follicle) but also from nevi and the eye. Most attention has however been directed to the loss of the easily detected dopa-positive (i.e., tyrosinase-positive) melanocytes, though it has been inferred that total graying involves also loss of at least some melanocytes from the stem cell reservoirs or at least the optimal proliferation and migration of progeny melanocytes.

The rate of melanocyte loss may vary between different body sites and within a compartment of the same body site. This may however reflect variations in the seeding of these compartments with melanocyte stem cells during embryogenesis. This heterogeneity may also reflect differences in their intrinsic 'melanogenetic clocks' or rather a deficit or loss of permissive microenvironments. Thus, loss of pigment is rather gradual in the epidermis, while age-related loss in the color of hair can be much more dramatic. A polygenic heredity appears to be a dominant factor in loss of hair pigmentation, as much of canities appears to be inherited in an autosomal dominant manner. The latter would explain the fact that entire extended families can experience marked early graying or conversely unusually late graying. There also may be some variation in the size of melanocyte reservoirs in different individuals, which may reflect different rates of 'seeding' from the neural crest during melanocyte precursor migrations during embryogenesis. There are several congenital conditions characterized by aberrant migration of melanocyte precursors along stereotypic routes, e.g., piebaldism.[46]

There is still a relative dearth of data on the mechanisms underlying melanocyte loss from the hair follicle with age.[47] It is likely that the hair cycle exerts significant effects, which may explain observed differences in the aging of these two skin melanocyte subpopulations. In that regard, the recent formal identification of melanocyte stem cells in the upper hair follicle[23,48] of mice will shed some light on the fate of their progeny in the epidermal and follicular melanin units during life in humans. One significant view to emerge is that melanocyte aging related to hair graying may involve defective maintenance of melanocyte stem cells. If the anti-apoptotic protein Bcl2 is deficient, loss of melanocyte stem cell is accelerated via apoptosis of these cells when they enter their dormant telogen state.[48] Nishimura and co-workers also suggested that physiologic aging of melanocyte stem cells may lead to the presence of ectopic pigmentation (or differentiation) within the stem compartment. Deficiency in the melanocyte master transcriptional regulator Mitf may also be involved in this process. More recently, this view was developed further by the same group of researchers with the demonstration in mice that accumulation of irreparable DNA damage with age (e.g., by ionizing radiation) may block melanocyte stem cell renewal. In fact, this study showed that this stress drives the stem cell instead to differentiate and thereafter to be lost from the niche rather than inducing its senescence or death. A potential role for ataxia-telangiectasia mutated (ATM) as a checkpoint molecule for stemness was suggested. The net effect is a deficiency of melanocyte stem cells needed to replace differentiated melanocytes lost from the catagen hair bulb upon the onset of new anagen phase.

Manipulation of hair follicle melanocytes *in vitro* was a long-sought aim in order to examine how these particular melanocytes behave and how they may age, as in canities. Epidermal and hair follicle melanocyte culture methodologies[26,49] provide a useful and accessible tool to examine differences in the aging of these closely related skin melanocyte subpopulations in humans. Primary culture revealed greater proliferation in the more immature melanocytes (i.e., less differentiated cells), and these proliferated even more than for matched epidermal melanocytes. As for other skin cells in culture, the loss of melanocyte-replicative potential *in vitro* is associated not only with increasing age of the donor, but there may also be an implication for how these melanocytes process melanin as they age *in vitro*. For example, the long-term continuous exposure of melanocytes to cAMP inducers (e.g., cholera toxin) induces both pigment production and a so-called 'pre-senescent' stage. Importantly, this signaling also does not directly stimulate the MC1-R as would be expected to occur more naturally.

The accumulation of cAMP in the cells in this way (i.e., by inhibiting the usual phosphodiesterase-associated breakdown of cAMP) also causes melanocytes to become large, epitheloid and stellate. A similar effect was observed when epidermal melanocytes were incubated with high levels of L-tyrosine (a melanin precursor). Here mitosis continued only in nonresponsive amelanotic cells, but proliferation was blocked in melanocytes that showed "pre-senescent" pigmented morphology.[47] Further studies revealed that the MAP kinase pathway was resistant to activation in these 'pre-senescent' melanocytes, resulting in their failure to proliferate.[50] Other features of these postmitotic melanocytes included increased expression of cyclin-dependent kinase (CDK) inhibitors (e.g., p21 and p16), and their binding to CDK4, which inhibit cell cycling.[51] Other features of replicative senescence in normal melanocytes included increased binding of CDK-I p16 (INK4a) to CDK4, down-regulation of cyclin E (and so loss of cyclin E/CDK2 activity), retinoblastoma protein RB under-phosphorylation (and so increased levels of E2F4-RB-repressive complexes) and progressive telomere shortening.[52,53] CDK- p21(Waf-1) and p27(Kip-1) are down-regulated, in contrast to the situation in fibroblasts. That these changes are not seen when melanocytes are induced to overexpress the catalytic subunit of the enzyme telomerase (hTERT) suggests they are indeed important for replicative senescence. How important this melanocyte-associated deviation from the molecular mechanisms found in fibroblasts is, is not yet clear, though it is likely to reflect the very different microenvironments of these two cutaneous cell types. For example, they are more likely to have different requirements to regulate the cell cycle in response to telomere attrition and thus prevent transformation.

The above observations have led to several theories of age-related change in melanocytes. The dominant theory 'borrowed' from mainstream aging research is the "free radical" theory of aging,[54,55] where the accumulation of oxidative damage determines the rate of aging. However, like several aging theories, it is not at all clear whether they adequately address the primary cause(s) of aging. For example, a failing melanocyte could be expected to exhibit a raft of 'free-radical'-associated anomalies, though these *per se* may not have set the cell on the road to degeneration.

ROS can damage biomolecules, and their effects on DNA (nuclear and mitochondrial) can be particularly devastating in that these lesions can drive an accumulation of mutations. However, cells are usually equipped with significant resources to combat the effects of oxidative stress, for example by a robust stimulation of antioxidant mechanisms. Aging impairs the cell's ability to mount a robust antioxidant response, such that increasing impairment with age can lead to uncontrolled damage to the melanocyte itself. The situation for melanocytes may in this regard be worse than that for other skin cells, given that much of the biochemistry of differentiated melanocytes is given over to the complex biochemical pathway of melanogenesis replete with oxidative stress generation, e.g., quinone and semi-quinone production.[25,56]

A recent study by the Peter's lab in Berlin has reported that the follicular melanin unit of graying hair follicles is indeed associated with increased melanocyte death by apoptosis and oxidative stress.[57] This study found that the "common" deletion in mitochondrial DNA (a marker of oxidative stress) occurred more prominently in graying hair follicles than in matched normally pigmented hair follicles. The interpretation here is that graying hair follicles are less well equipped to handle exogenous oxidative stress and that this was likely due to their impaired antioxidant systems. We need to be a little careful how we interpret such findings, however, as hair fibers in gray/white hair follicles can grow even faster than those in matched pigmented hair follicles both *in vitro*[57] and *in situ*.[30,50,58] This may infer that active keratinocyte proliferation is facilitated in this microenvironment and that these cells may either express intact antioxidant systems or are more resilient in the presence of oxidative stress than melanocytes. A recent study by Schallreuter's laboratory in Bradford used FT-Raman spectroscopy in vivo to demonstrate that human gray/white scalp hair shafts accumulate hydrogen peroxide in millimolar concentrations.[33] That this tiny molecule is not rapidly lost from the fiber suggests that it may rather be trapped somehow in the hair. The associated scalp tissue also revealed little catalase and methionine sulfoxide reductase A and B protein expression, and there was a functional loss of methionine sulfoxide (Met-S = O) repair in the affected hair follicle. This environment damages tyrosinase function. However, it is not yet clear whether these changes are causative in the loss of hair pigment in graying hair follicles. They could rather be a secondary effect of canities.

### Is the hair pigmentary unit a victim of its melanogenic prowess?

**Figure 1:**
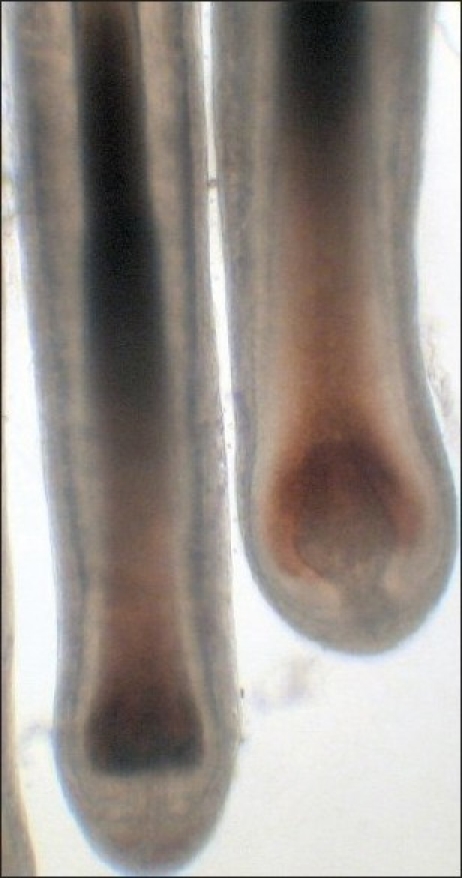
Fully-pigmented human anagen scalp hair follices showing intense melanization (brown and red) of the hair bulb and hair shaft. Whole mount (i.e, entire follicle), bright-field light microscopy

Hair bulb melanocytes during anagen exhibit both a phenomenally high capacity for melanin production and a very high intracellular melanin load. In this way, follicular melanocytes differ markedly from other cutaneous melanocytes. Indeed, a relatively small number of melanocytes, perhaps as few as 100 cells per human scalp anagen hair follicle, can, in a single hair growth cycle, produce enough melanin to intensely pigment hair fibers of up to 1.5 m in length [Figure 1]. Recently an assessment of the anagen-block hair follicles as seen in exceptional cases of long scalp hair (i.e., up to 4.6 m during an anagen of over 26 years[7]) suggests that these cells can maintain this very high melanin synthesis rate even longer provided they reside in an anagen-associated permissive microenvironment.

It is not clear why the cytoplasm of bulbar melanocytes should contain a much higher fraction of mature melanosomes than occurs in epidermal melanocytes, particularly in dark-haired Caucasians. There is no evidence that melanin transfer to surrounding keratinocytes is more efficient (i.e., higher rate of melanin donation to keratinocytes) in the epidermis. Indeed the 1-1.5 cm/month production of pigmented hair fiber is as impressive, if not more so, as the monthly turnover of the much thinner human epidermis. However, the prolonged melanogenesis characteristic of hair bulb melanocytes during anagen is likely to generate large amounts of ROS via the oxidation of tyrosine and dopa to melanin.[5,59] If these ROS are not efficiently removed, the resultant oxidative stress would damage the melanocyte itself and pose significant potential risks for mutation. This could be prevented if melanogenic bulbar melanocytes assumed a postmitotic, terminally differentiated '(pre)senescence' status.

## HISTOPATHOLOGY OF HAIR GRAYING (CANITIES)

**Figure 2:**
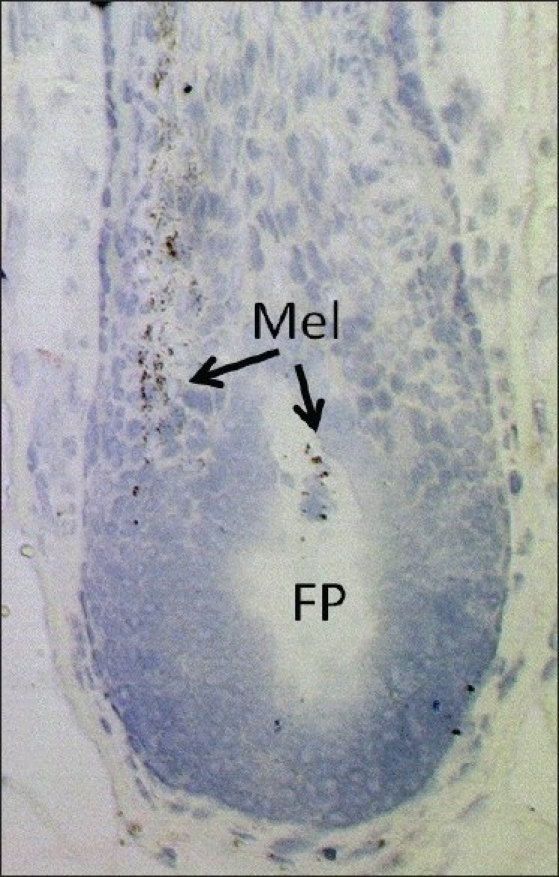
Loss of melanocytes from the hair bulb of aging human anagen scalp hair follicles. FP = Follicular papilla, Mel = Melanin. Bright-field light microscopy with tolouidine blue staining

Gray hair follicles have markedly reduced numbers of differentiated and functioning melanocytes located in the hair bulb; while hair follicles described as 'senile white' follicles may have none at all in the hair bulb region of the hair follicle. The focus here is therefore on the hair bulb, though it is clear that some (mostly immature) melanocytes can be retained for some time after graying begins elsewhere in the hair follicle. Exactly when so-called white hair follicle contains no melanocytes of any kind throughout the entire hair follicle remains open in my view, despite some more established positions emerging in the literature.[2‐4,60] The precise mechanisms responsible for the loss of melanogenically active melanocytes from anagen adult hair follicles with increasing age remain rather speculative. In true gray hair follicles, the melanin granules can be readily detected within the pre-cortex and hair fiber in an often asymmetric pattern [Figure 2]. Gray hair bulbs exhibit a much reduced, yet detectable, dopa-oxidase reaction, indicating that melanocytes remain with at least some tyrosinase activity. Given the high level of accessibility of skin for study of aging, gray hair follicles are a potentially interesting test system to examine aging of melanocytes, and even as neural cell proxies. The process of melanosome transfer from the residual and degenerating melanocytes in these affected hair bulbs is also a fascinating model for examining how defective cell cytoplasmic process and dendrites are involved in melanosome transfer in the graying hair follicle. It is particularly interesting to note that pre-cortical keratinocytes surrounding these failing melanocytes fail to receive melanin granules from these damaged melanocytes despite the latter cells still containing significant levels of melanin.[2‐4] In this sense, the normal relationship of melanocyte to keratinocyte in the 'follicular melanin unit' breaks down.

Canities as a true aging process is supported by the somewhat 'messy' nature of follicular melanin unit degeneration. Significant melanin debris/incontinence can be seen within and around graying hair bulbs, and at a level much greater than commonly seen during the more programmed breakdown of the follicular melanin unit during the catagen phase of the hair growth cycle.[2‐4] Degenerating bulb melanocytes in graying follicles may exist in this dystrophic hypertrophic state for some time. When the intracellular environment of these degenerating melanocytes is examined, it is clear that the sub-cellular compartmentalization of melanogenesis has become dysregulated. This can be seen in the packaging of melanosomes within auto-phagolysosomes, suggesting that the defective cells are attempting to remove aberrant (leaky?) melanosomes. If these melanin-producing melanocyte-specific organelles indeed become leaky, these melanosomes may release reactive oxygen metabolites derived from the melanin bio-synthetic pathway into the cell cytoplasm and trigger autophago-lysosomal degradation. Thus, if the cell is unable to efficiently remove defective cell constituents like melanosomes, the cell itself may become compromised and die.[61] Evidence of melanocyte death in canities can be seen by levels of ectopic melanin redistributed to the follicular papilla and/or connective tissue sheath of hair follicles that lack any evidence of intact melanocytes or active melanogenesis. The most reasonable interpretation of these findings is a very recent loss of melanin/melanocytes from a previously melanogenic hair follicle.

A defective compartmentalization of the biochemically highly reactive process of melanogenesis, together with a reduced or inefficient antioxidant system, may lead to increased availability of ROS in the melanocytes. Further support for the involvement of ROS in the histopathology of canities is suggested by the observation that melanocytes in graying and white hair bulbs may be vacuolated, a common cellular response to increased oxidative stress. Indeed, there is a correlate here with another pigmentary anomaly, i.e., loss of functional epidermal melanocytes in vitiligo. In the latter, millimolar levels of the oxidant H^2^O^2^ can be found, which damages the epidermal melanocytes.[62] Recent studies from the same research group reported the presence of millimolar H_2_O_2_ within gray and white hair fibers but not in pigmented fibers.[33] This study did not comment on tissue levels of this pro-oxidant however. However, unlike in vitiligo, the loss of bulbar melanocytes in canities does not appear to trigger an immune system response. Rather, in canities melanocyte degeneration leads to the removal of degenerative melanogenic melanocytes from the hair bulb of graying and white hair follicles. This 'clean-up' process may be associated however, with an increase in the number of dendritic cells, including Langerhans cells,[63] attracted to this immune-privileged part of the hair follicle[64] by stimulation from degenerating canities-affected melanocytes.

### Does melanocyte loss from aging hair follicles affect their growth behavior?

Melanin production by melanocytes is a much more limited activity than is keratin production by the hair follicle keratinocytes. The latter has been shown to continue successfully for over 100 years without fail for long-living individuals, a feat which may infer a greater integrity of both keratinocyte replacement and keratin production. Moreover, there appear to be significant regional variations in the progression of melanocyte loss throughout the body. In general this effect is more striking on the scalp. Moreover, there may be some region-specific differences in the handling of stress by melanocytes and melanocyte stem cells in some hair follicle types. In this regard, it is of note that whereas scalp hair follicles gray early, hair follicles of the eyebrow and eyelash gray very slowly even in the same scalp-gray-haired individual. This may be related to differences in ROS-handling apparatus in these different hair follicle types, or that these hair follicle types are bestowed with different levels of stem cells. Moreover, while scalp hair bulb melanocytes express undetectable levels of dopachrome tautomerase, similar melanocytes in eyebrow follicles express this melanogenic and oxidative stress-protective enzyme.[58,65,66]

As mentioned above, the hair follicle pigmentary unit is maximally functioning during our post-adolescence and early adulthood, when terminal hair growth is optimal and hair color has settled to its preferred tonal variant, i.e., when fully responsive to the post-puberty hormonal stimulus. This age group has scalp follicular melanin units that are only a few hair growth cycles old. Given an average hair cycle period of 3.5 years, some individual scalp hair follicles will experience fewer than 10 melanocyte re-seedings from the presumptive reservoir in the average "gray-free" lifespan of 35 to 40 years for Caucasians.[67]

The onset and progression of graying or canities correlate very closely with chronological aging (but not with photo-aging) and occur in varying degrees in all individuals eventually, regardless of gender or race. The age of onset is genetically controlled and heritable, such that on an average Caucasians begin to gray in their mid-30s; Asians, in their late-30s; and Africans, latest in their mid-40s. Indeed, hair is said to gray prematurely only if graying occurs before the age of 20 years in whites, before 25 years in Asians and before 30 years in Africans. Although not formally tested, a good rule of thumb is that by 50 years, 50% of people have 50% gray hair.[21] Even the term *gray* can be controversial for some who consider that this color is derived from an admixture of fully white and fully pigmented hair. The implication here is that unpigmented hair emerges from the surface of the epidermis already as a white hair fiber or that the hairs are demarcated as two-tone rather than with color dilution. This author has often observed canities to affect individual hair follicles during a single anagen VI growth phase, such that there is a gradual loss of pigment along the same hair shaft. However, there is more to our perception of gray/white hair than just an absence of melanin. The hair fiber is a complex interplay of many physical characteristics including color, hair fiber geometry and curvature that can determine shine and luster. Thus, the perception of graying can vary significantly in the general population, with early graying being first noticeable in dark-haired individuals. Paradoxically however, graying can be more extensive in these dark-haired individuals before they reach the blanched effect; the reverse is true for blond hair. The rate of graying is also highly variable, not only on different areas of the scalp but also across the body. This may reflect variations in original melanocyte precursor seedings during melanoblast migrations in embryogenesis or in differences of niche quality. Thus, scalp hair graying first appears usually at the temples and spreads to the vertex and then the remainder of the scalp, affecting the occiput last. Beard and body hair are usually only affected later.

Hair bulb melanocytes and pre-cortical keratinocytes are the interactive elements of the follicular melanin unit.[13,21,24] There is currently much interest in dissecting the nature of this interaction, with *in vitro* evidence from mixed-origin co-cultures suggesting that the keratinocyte directs the melanocyte to produce the type of melanin matching the keratinocyte donor's skin phototype. Moreover, melanin transfer to the keratinocyte partner appears to reduce the latter's proliferative potential and rather may stimulate its terminal differentiation. Thus, it is perhaps to be expected that pre-cortical keratinocyte behavior may change in the absence of melanocyte influence. There is some clinical evidence for this: White beard hair appears to grow faster than adjacent pigmented hair *in vivo*,[59] and white hair follicles exhibit a higher rate of hair fiber elongation *in vitro* than do matched pigmented hair follicles.[57] In this context, melanosomes may therefore act as regulatory packages,[68] for example by providing a buffer for calcium, with implications for second messenger/cell signaling in melanogenesis, melanosome transfer and keratinocyte differentiation. Furthermore, the saturation binding of transition metals (e.g., iron, copper) to melanin is also likely to influence the antioxidant defense of the melanosomereceiving keratinocyte.[3‐5] Moreover, melanocytes as producers of a range of bio-response modifiers (e.g., cytokines, growth factors, eicosanoids, adhesion molecules and extracellular matrix) can influence the behavior of neighboring keratinocytes.[69,70]

Evidence of melanocyte-keratinocyte interactivity can also be perceived clinically in the hair fiber too, with graying hair commonly coarser, wirier and more unmanageable than its pigmented equivalent. Here too, the absence of melanin reflects a change in the chemical and physical properties of the post-pigmented hair fiber.[30] Additionally, gray hair is often unable to hold a set and is more resistant to incorporating artificial color. These changes have significant implications for the cosmetics industry. On the basis of the above findings, it appears that graying hair follicles may reprogram their matrix keratinocytes to increase the production of medullary, rather than pre-cortical, keratinocytes.

## CONCLUSION

**Figure 3:**
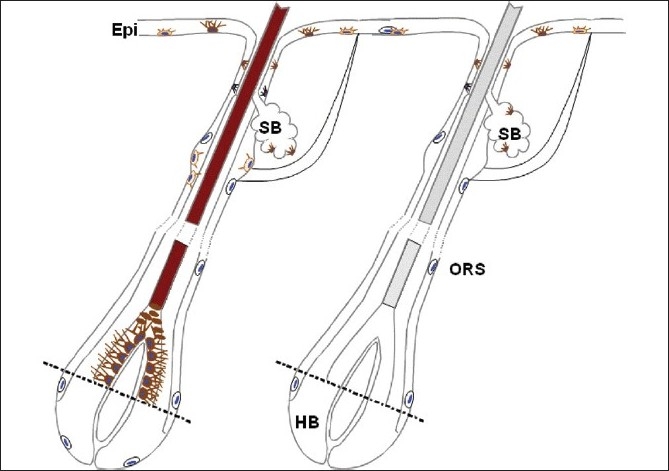
Cartoon of pigmented and canities-affected human anagen scalp hair follicle, showing loss of melanization in the hair bulb and hair shaft with graying. Some amelanotic melnocytes can be seen in the outer root sheath (ORS) and in the most proximal and peripheral hair bulb (HB). SB - Sebaceous gland; Epi - Epidermis

Progress in the study of the regulation of human hair pigmentation and associated changes with aging has only recently been the focus of attention, after the initial excellent work of Fitzpatrick and co-workers.[12]This was in part due to an over-reliance on the murine systems. An emerging view is that mouse-human species-specific differences (at least for pigmentation research) can be quite marked. One particular fruitful topic for future study will be to elucidate the function of the amelanotic melanocytes distributed in the outer root sheath of human scalp hair follicles[Figure 3]. It will be important to determine if these cells are indeed progeny of so-called stem cells from the bulge area of the hair follicle, and if so, whether they retain some stem cell characteristics themselves. The recruitment of these immature outer root sheath melanocytes for re-pigmentation of the hair follicle (and even the overlying epidermis, especially after wounding[70]) may offer significant clinical gains. Alternatively, these nonpigmenting follicular melanocytes may represent a subpopulation of transient or migrating melanocytes that can only differentiate when in a permissive microenvironment, e.g., melanogenic zone close to the follicular dermal papilla. The reversal of canities after some types of therapy, e.g., radiation/drug, may involve a cytokine-induced activation of these outer root sheath melanocytes. The future looks very bright and potentially colorful for hair pigmentation research.
